# Specific expression and export of the *Plasmodium falciparum* Gametocyte EXported Protein-5 marks the gametocyte ring stage

**DOI:** 10.1186/s12936-015-0853-6

**Published:** 2015-08-28

**Authors:** Marta Tibúrcio, Matthew W. A. Dixon, Oliver Looker, Sumera Younis Younis, Leann Tilley, Pietro Alano

**Affiliations:** Dipartimento di Malattie Infettive, Parassitarie e Immunomediate, Istituto Superiore di Sanità, Viale Regina Elena 299, 00161 Rome, Italy; Department of Biochemistry and Molecular Biology, Bio21 Molecular Science and Biotechnology Institute, The University of Melbourne, Melbourne, VIC Australia; The Francis Crick Institute, Mill Hill Laboratory, The Ridgeway, Mill Hill, London, NW7 1AA UK; Department of Parasitology, Biomedical Primate Research Centre, PO Box 306, 2280 GH Rijswijk, The Netherlands

**Keywords:** *Plasmodium*, Gametocyte, Exported protein, Chromosome 9, Commitment, Sexual differentiation, Early gametocyte marker

## Abstract

**Background:**

*Plasmodium falciparum* sexual development plays a fundamental role in the transmission and spread of malaria. The ability to generate gametocytes can be lost during culture in vitro, often associated with the loss of a subtelomeric region of chromosome 9. Gametocytogenesis starts with erythrocyte invasion by a sexually committed merozoite, but the first available specific marker of sexual differentiation appears only from 24 h post invasion.

**Methods:**

Specific antibodies and gene fusions were produced to study the timing of expression and the sub-cellular localization of the *P. falciparum* Gametocyte EXported Protein-5 (PfGEXP5), encoded in the subtelomeric region of chromosome 9. Expression patterns were examined in wild-type parasites and in parasite lines mutated in the Apetala2-G (AP2-G) transcription factor, governing a cascade of early sexual stage specific genes.

**Results:**

PfGEXP5 is highly expressed in early sexual stages and it is actively exported to the infected erythrocyte cytoplasm from as early as 14 h post-invasion in haemozoin-free, ring stage-like parasites. The pattern of PfGEXP5 expression and export is similar in wild-type parasites and in independent AP2-G defective parasite lines unable to produce gametocytes.

**Conclusions:**

PfGEXP5 represents the earliest post-invasion sexual stage marker described to date. This provides a tool that can be used to identify sexually committed ring stage parasites in natural infections. This early gametocyte marker would enable the identification and mapping of malaria transmission reservoirs in human populations and the study of gametocyte sequestration dynamics in infected individuals. The fact that regulation of PfGEXP5 does not depend on the AP2-G master regulator of parasite sexual development suggests that, after sexual commitment, differentiation progresses through multiple checkpoints in the early phase of gametocytogenesis.

**Electronic supplementary material:**

The online version of this article (doi:10.1186/s12936-015-0853-6) contains supplementary material, which is available to authorized users.

## Background

Malaria is a vector-borne disease caused by the genus *Plasmodium*. It is responsible for an estimated 200 million clinical cases and approximately 580,000 deaths per year, mostly in sub-Saharan Africa [1], particularly affecting pregnant women and children. Of the five malaria species that infect humans, *Plasmodium falciparum* is the most virulent, responsible for 90 % of disease cases [2].

Disease is associated with a 44–48 h cycle of asexual replication that takes place within the red blood cells (RBCs) of the human host, where *P. falciparum* matures through the ring, trophozoite and schizont stages. Clinical symptoms range from uncomplicated fevers to life-threatening cerebral and placental malaria [3]. During asexual cycling a portion of the parasite population commits to sexual differentiation (gametocytogenesis), thus ensuring parasite transfer to the transmission vector, an *Anopheles* mosquito [4].

Parasite sexual commitment occurs in the asexual generation preceding gametocytogenesis, where each individual schizont produces a progeny of merozoites that uniformly develop into either asexual parasites or sexual parasites of the same sex [5–7]. It is currently debated whether in natural infections a constant fraction of parasites converts to sexual development during each asexual cycle or whether commitment is an environment-sensitive, triggered event [8].

After invasion, a sexually committed parasite develops intra-erythrocytically over a period of ten to 12 days, progressing through five distinct stages of gametocyte development [9, 10]. In the earliest stage of sexual development, the intra-erythrocytic parasite is morphologically indistinguishable from an asexual ring-stage parasite, and no specific markers are currently available to identify this ‘sexual ring’ stage. At 24–30 h post invasion (pi), about the time an asexual parasite enters the trophozoite stage, the early gametocyte (stage Ia) starts to produce the early stage-specific proteins Pfs16 and Pfg27 [11–15]. At about 48 h pi, ultrastructural features of the sexual parasite first become apparent (stage Ib to stage II), such as the assembly of a bilamellar membrane structure subtended by a network of subpellicular microtubules [16, 17].

In natural infections, stages Ib to IV gametocytes are sequestered in deep tissues, such as the bone marrow [18, 19]. The only morphologically recognizable gametocyte stage that is visible in the peripheral circulation is stage V. Following ingestion by a mosquito, these mature gametocytes are responsive to the triggers that initiate gamete formation, the first step in transmission to the insect vector [4, 20]. In contrast, the lack of specific markers for sexually committed parasites at the ring stage makes it impossible to answer the fundamental question of whether the earliest gametocyte stages (i.e., before 24 h pi) are formed, and remain, within the deep tissues or are freely circulating [18, 21]. In addition, the lack of a sexual ring-stage diagnostic reagent makes it difficult to address key epidemiological questions relating to the dynamics of parasite transmission. For example, the identification of the human malaria reservoirs, such as long-term carriage of infective sub-microscopic gametocytes in asymptomatic individuals, would be facilitated by a ring stage marker [21]. Similarly, early detection of gametocytes would be important to evaluate the effects of different treatment regimens of symptomatic individuals on gametocyte carriage [22, 23]. Both of these questions have important implications for malaria control.

The ability of parasite lines and clones to generate gametocytes can be lost during culture in vitro. In *P. falciparum*, the gametocyteless clone F12 was derived from a gametocyte-producing parental 3D7 clone in a controlled 18-month asexual propagation experiment [24]. F12 parasites are unable to produce morphologically recognizable gametocytes, nor to express the stage I marker Pfg27 [24]. Exon sequencing of the genomes of F12 and of independently obtained gametocyteless parasites, such as GNP-A4, was used to identify the genetic defect [25]. Distinct null mutations in the coding sequence of a putative transcription factor of the *P. falciparum* Apetala2 family member, PfAP2-G, were shown to be associated with defective gametocyte production [25]. Gene disruption and down-regulation of the PfAP2-G expression functionally confirmed the role of this protein. PfAP2-G appears to be responsible for switching on the expression of a small set of early gametocyte proteins, which leads to the appearance of morphologically recognizable gametocytes [25].

The loss of gametocyte production has also been observed in parasite lines with different genetic backgrounds. For example the C10 parasite line, which is derived from isolate 1776 [26] produces no morphologically distinguishable gametocytes [24]. In this and in other lines of different geographical origin, the loss or dramatic reduction of gametocyte production is associated with subtelomeric deletions of 100–150 kb near the right end of chromosome 9 [24, 27], strongly suggesting that this region encodes another major genetic determinant of gametocytogenesis.

A genome-wide expression analysis identified a suite of genes that are activated early in gametocyte development [28] and a proteomics analysis revealed that a group of putatively exported proteins are over-represented in these stages [29]. These proteins were designated as *P. falciparum* Gametocyte EXported Proteins (PfGEXP) and the export of some of these proteins has been demonstrated, including Pfg744 [30], PfGECO [31] and PfGEXP10 [29].

*Plasmodium falciparum* Gametocyte EXported Protein-5 (PfGEXP5) is a member of this family. This work shows that PfGEXP5 is exported into the host cell cytoplasm from as early as 14 h after invasion of a sexually committed merozoite, making it the earliest gametocyte-specific marker so far described. It also shows that PfGEXP5 is expressed and exported upon exposure of the F12 and GNP-A4 parasite clones to gametocyte-inducing conditions, indicating that this early gametocyte protein functions independently of the developmental switch governed by the PfAP2-G transcription factor.

## Methods

### Parasite culture

A high gametocyte-producing *P. falciparum* line 3D7 [32] was cultured in human 0+ RBCs, sourced from Prof G. Girelli, Dipartimento Biopatologia Umana, University of Rome ‘La Sapienza’ or from the Australian Red Cross Blood Service. Parasites were cultured in RPMI-HEPES supplemented with 5–10 % human serum with-without 0.25 % AlbuMAX II. Transfected cultures were maintained in media supplemented with 5 nM WR99210. Parasite growth was monitored by Giemsa-stained thin blood smears. Initial synchronization of the ring stages was performed by addition of 5 % sorbitol. For tight synchronization very late stage schizonts were purified by magnetic separation (CS columns; Miltenyi Biotec) [33] and mixed with uninfected RBCs and harvested 2 h after invasion as described previously [34]. Gametocyte cultures of the desired stage were obtained as described in [29] or using a method adapted from Fivelman et al. [35] as described in Dearnley et al. [17].

### Production of the PfGEXP5-GST fusion proteins and mouse immunization

A 483 bp *pfGEXP05* (PF3D7_0936600) fragment from the intron-less genomic region, encoding amino acids 104–264 of the predicted protein sequence, was cloned in a *Bam*HI/*Not*I-digested pGEX-6P-3 vector and used to produce a GST fusion protein in *Escherichia coli* BL21 (2 h at 37 °C), using previously described methods [36]. The protein was solubilized in 6 M urea followed by SDS-PAGE, the electro-eluted fusion protein was dialyzed and used for immunizing mice as described in [29].

### Production of PfGEXP5-GFP fusion protein and parasite transfection

A full length PfGEXP5 was PCR amplified from 3D7 cDNA using the primers PfGEXP5-GFPF (**agatct**atgaaagatcagattgaa) and R (**ctgcag**tcccatatgtctaggtgt) and directionally cloned into the *Bgl*II and *Pst*I restriction sites (Bold) of the pCGFP-Entry vector to yield the pCGEXP5-GFP-Entry vector. The CRT promoter sequence from this vector was replaced with 1500 bp of sequence from the 5′ UTR of PfGEXP5. The promoter sequence from the 5′ UTR was PCR amplified from genomic 3D7 using the primers GEXP5-ProF (**gtcgac**ggtggaagatcatttact) and GEXP5-ProR (**agatct**tctaaaaaatataaaaataaa) and was directionally cloned into the pC70GFP-Entry vector pre-digested with *Sal*I and *Bgl*II to excise the CRT promoter sequence. The resultant vector contained the full length cDNA sequence and 1500 bp of the 5′ UTR of the native PfGEXP5 locus. This pPfGEXP5-GFP-Entry vector was then recombined with the pHH1-Destination vector as per the manufacturer instructions. The resultant vector pHH1-PfGEXP5-GFP (PfGEXP5-GFP) and transfections were performed as previously described [37].

### Western blot analysis

Protein extracts from Percoll-purified gametocytes from day 2 post induction were obtained and lysed by Equinatoxin II (EqtII; [38]). Soluble and insoluble fractions were separated by SDS-PAGE, transferred to nitrocellulose filters. Primary and secondary antibodies were prepared in 3.5 % skim milk in PBS and all washing steps were performed in PBS containing 0.02 % Tween 20. The following primary antibodies were used: mouse anti-PfGEXP5 (1:200), mouse anti-Pfs16 (1:1000, [39]) or rabbit anti-GAPDH (1:5000) [40]. Primary antibodies were added to blots and incubated overnight at 4 °C. Anti-mouse and anti-rabbit antibodies (1:20,000) conjugated to horseradish peroxidase (HRP) were incubated for 1 h at room temperature, prior to detection by chemiluminescence.

### Immunofluorescence analysis

Parasites were fixed in suspension with 4 % paraformaldehyde, 0.075 % glutaraldehyde and permeabilized with 0.1 % Triton X-100 [41]. The antibodies used were: mouse anti-PfGEXP5 (1:100), rabbit anti-Pfg27 (1:200), mouse anti-Pfs16 (1:500) [39, 42], rabbit anti-RESA (1:200) [43], rabbit anti-KAHRP [44] and mouse/rabbit anti-GFP (1:200) (Roche) (rabbit [45]). Anti-mouse and rabbit secondary antibodies conjugated to fluorescein/Alexa 488, rhodamine/Alexa568 or Alexa 647. Images were collected using a Leica DMR fluorescence microscope fitted with a Leica cooled CCD camera, and deconvolved using the LAS V3.8.0 (Leica Application Suite 3.8.0) software, or with a Delta Vision Elite wide field deconvolution microscope. Images were processed with ImageJ [46].

## Results

### PfGEXP5 is expressed in early stage gametocytes and exported to the host cell cytoplasm

**Fig. 1:**
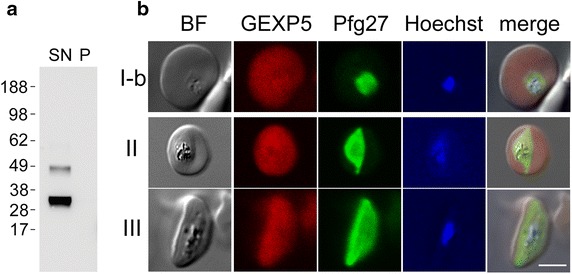
PfGEXP5 protein expression and export in gametocyte development. **a** Western blot analysis of an Equinatoxin-II extract (SN) and pellet (P) of *P. falciparum* stage III gametocytes, probed with the anti-PfGEXP5 antiserum. **b** Immunofluorescence microscopy of different stages of gametocyte maturation (3D7). Cells were labelled with mouse anti-PfGEXP5 (*red*) and rabbit anti-Pfg27 (*green*). Nuclei were stained by Hoechst. PfGEXP5 is located in the RBC cytoplasm. *Scale bar* 5 μm

The two-exon gene *pfgexp5* (PF3D7_0936600) encodes a protein with a predicted molecular mass of 34,909 Da. The sequence includes a predicted protein export element/host targeting signal (PEXEL/HT) motif, which indicates export to the host RBC [47], and a conserved PHIST-C domain [48]. To characterize the expression pattern and cellular location of PfGEXP5, antibodies were generated against a recombinant fragment (amino acids 104–264) fused to GST. To examine the reactivity against sexual stage parasites, a culture of gametocyte-producing 3D7 line [32] was allowed to reach a high parasitaemia to induce sexual differentiation, and *N*-acetyl-glucosamine (Glc-NAc) was used to clear residual asexual parasites. The mouse antiserum was used to probe a Western blot of a sample of stage III gametocytes that had been treated with Equinatoxin-II (EqtII) [38] to selectively permeabilize the RBC membrane (Fig. 1a). The antiserum recognized a protein in the supernatant fraction, migrating with an apparent molecular mass of 33 kDa, which is slightly larger than the predicted size of the PEXEL-cleaved polypeptide. This is likely due to aberrant migration of some *Plasmodium* proteins, which is attributed to their unusual sequence composition [49, 50]. While a weakly cross-reacting band was occasionally observed in the infected RBC supernatant, no band were observed in the parasite pellet.

The above results indicate that PfGEXP5 is located in the infected RBC cytoplasm. This was directly tested using immunofluorescence microscopy of gametocytes at different stages of development from the genetically diverse parasite clones 3D7 and HB3. The samples were fixed with paraformaldehyde/glutaraldehyde, permeabilized with Triton X-100 and labelled with mouse anti-PfGEXP5 and rabbit anti-Pfg27 [15]. Pfg27 is a sexual stage marker expressed in the gametocyte cytoplasm and nucleus from 30 to 36 h pi and maintained during further development [14]. A signal for PfGEXP5 was observed from stage I of gametocyte development in both parasite lines (Fig. 1b; Additional file 1: Figure S1A). In contrast to the Pfg27-specific signal, marking the parasite cell, the PfGEXP5-specific fluorescence is clearly detectable in the RBC cytoplasm in all stages analysed, thus confirming the Western blot analysis of the selectively permeabilized cells. PfGEXP5 is maintained during gametocyte development to stage IV, then declines in stage V (see Additional file 1: Figure S1B). The same analysis was conducted with an anti-GST antibody, directed against the GST moiety of the PfGEXP5 fusion protein used to immunize mice. No staining could be observed with the anti-GST antibody (see Additional file 1: Figure S1A), thus confirming the specificity of the anti-PfGEXP5 antiserum.

In the course of these experiments it was noticed that PfGEXP5 is expressed in a population of parasites that do not produce Pfg27. The parasites exhibited ring-stage morphology, with no visible haemozoin crystals. This result strongly suggests that PfGEXP5 is expressed and exported in very early (ring stage) gametocytes, before the expression of Pfg27.

### PfGEXP5 is initially expressed around 14 h post invasion and exported to the RBC cytoplasm in parasites committed to sexual development

**Fig. 2:**
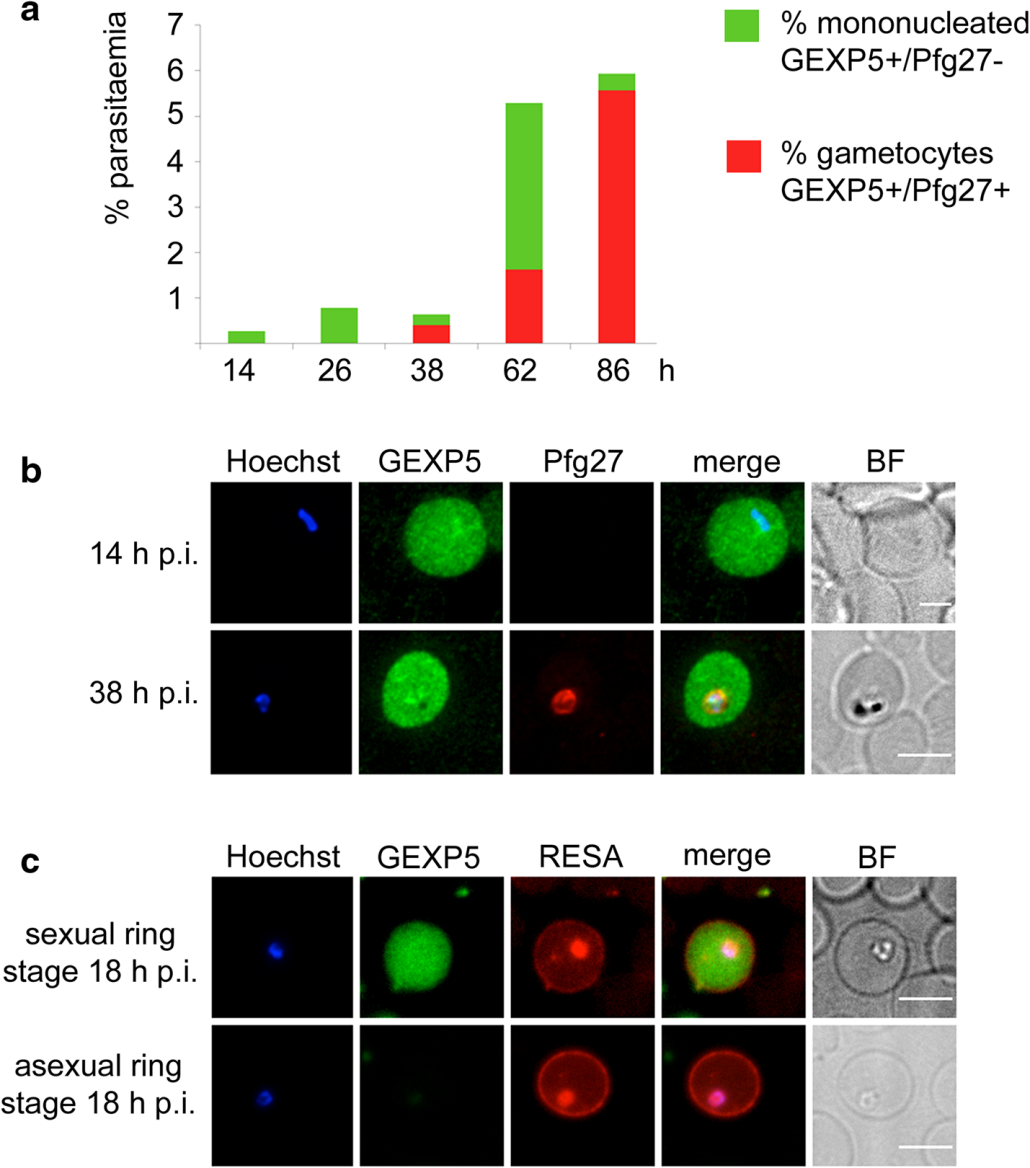
Time course analysis of PfGEXP5 expression. **a** Time course of antigen expression after gametocyte induction in a synchronous 3D7 culture. The percentage of mononucleated parasites that are positive for PfGEXP5 alone (*green*) or for both PfGEXP5-positive and Pfg27 (*red*) are shown at different time points. These data are typical of experiments performed three times. **b** Representative immunofluorescence images of (*top panel*) a ring stage-like parasite at 14 h pi positive for PfGEXP5 (*green*) and negative for Pfg27 (*red*), and (*lower panel*) a pigmented double-positive parasite at 38 h pi. **c** Immunofluorescence microscopy of ~18-h gametocyte and asexaul ring-infected RBCs labelled with anti-PfGEXP5 (*green*) and anti-RESA (*red*) antibodies. Brightfield images (BF) and nuclear staining are shown at *right* and *left*, respectively. *Scale bar* 5 μm

To investigate this point, a highly synchronous (2-h window) culture of 3D7 trophozoites was obtained. Magnet purified mature schizonts were incubated for 2 h with uninfected RBCs after which a sorbitol treatment was applied to obtain a culture containing only newly infected ring stages. Parasite development was followed over a 96-h period, after the first round of invasion (Fig. 2a). Immunofluorescence microscopy using antibodies against PfGEXP5 and Pfg27 showed that by 14–16 h pi, a sub-population of mononucleated parasites were positive for PfGEXP5 but negative for Pfg27, and had no detectable haemozoin pigment (Fig. 2b). By 38–40 h pi, 60 % of the PfGEXP5-positive parasites were also positive for Pfg27, which identified them as stage I gametocytes (Fig. 2b). This pattern of expression was confirmed in three independent experiments.

In an independent time-point experiment, parasites sampled at 14–18 h pi were dual labelled with antibodies against PfGEXP5 and the knob-associated, histidine-rich protein (KAHRP), a knob component appearing from 16 h pi in asexual parasites, and undetectable in the knobless stage I/early II gametocytes [51]. Failure to detect double positive parasites for such antisera shows that PfGEXP5 is not produced in asexual stages (see Additional file 1: Figure S2A). The same parasite sample at 14–18 h pi was dual labelled with antibodies against PfGEXP5 and the ring-infected erythrocyte surface antigen (RESA). RESA is one of the earliest known exported proteins in asexual parasites [52, 53]. It associates with the spectrin network underlying the RBC membrane, where it remains detectable until 18–24 h pi. A population of PfGEXP5-positive cells was observed that also exhibited RESA labelling at the RBC periphery. This was distinguishable from a population of infected RBCs where a similar RESA labelling profile was not associated with GEXP5 expression (Fig. 2c). These two cell types may represent ‘sexual ring’ and ‘asexual ring’ stages, respectively.

It was not possible to perform dual labelling of these samples with antibodies recognizing Pfs16, the earliest known gametocyte marker, due to the lack of a suitable rabbit antibody. Therefore the appearance of haemozoin was used as a surrogate marker of parasite age (post-invasion) of the above parasites. In asexual parasites, haemozoin is first visible at about 14 h pi [54]. Most (79 %) of the PfGEXP5-positive parasites exhibited no visible haemozoin crystals (e.g., Fig. 2b, c), indicating that haemoglobin digestion was minimal, while small haemozoin crystals were apparent in the other 21 %.

These experiments confirm that the anti-PfGEXP5 antiserum is able to recognize sexually committed parasites from as early as 14 h pi and provides the first evidence that RESA is expressed in early gametocytes.

### A PfGEXP5-GFP chimera is exported to the RBC cytoplasm

**Fig. 3:**
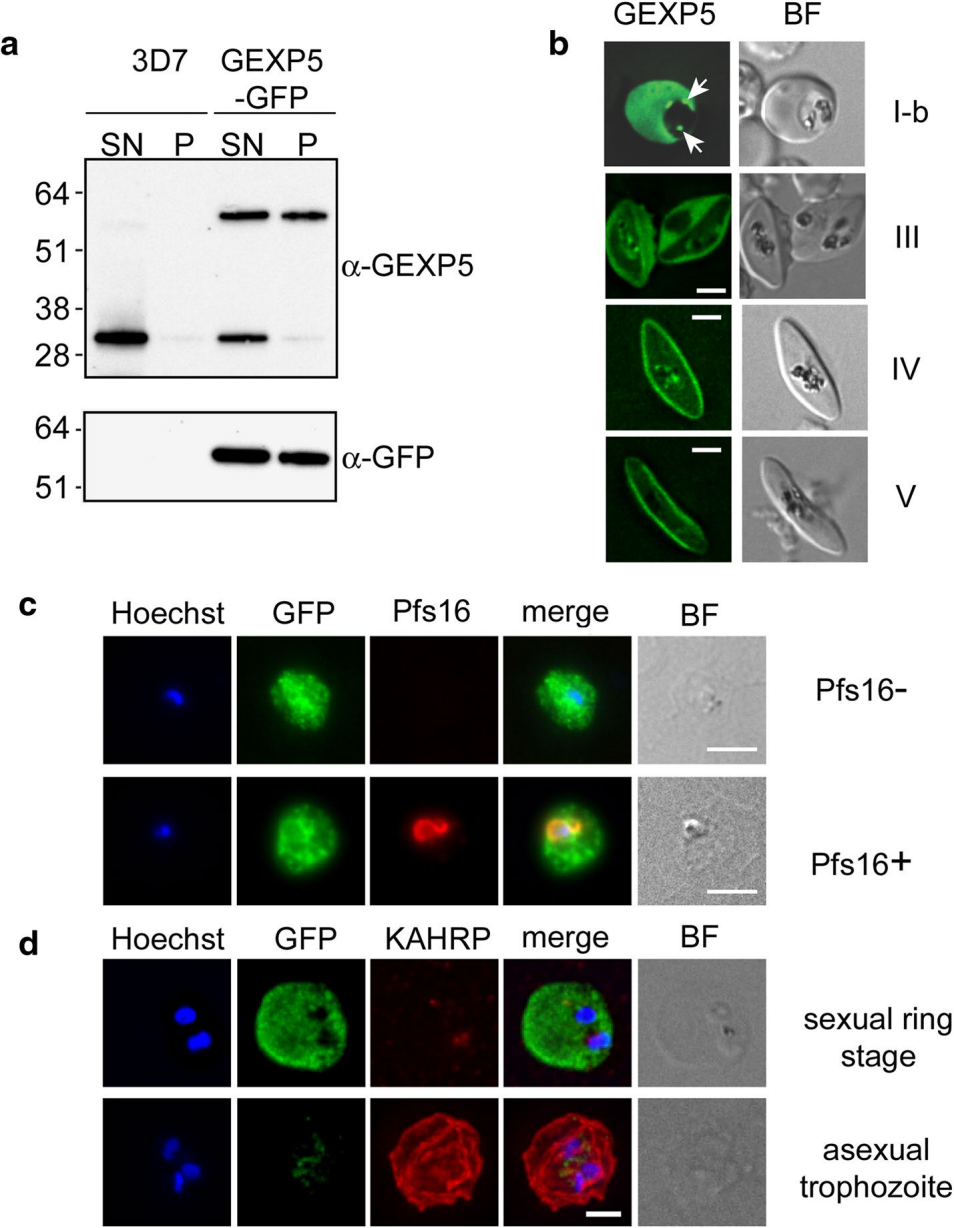
A PfGEXP5-GFP chimera is exported to the RBC cytoplasm. **a** Western blot analysis of the 3D7 parent line and the PfGEXP5-GFP transfectant. Stage III gametocytes were treated with Eqt-II and the pellet (P) and supernatant (SN) fractions subjected to Western blot and probed with anti-PfGEXP5 and anti-GFP antibodies. **b** Expression of PfGEXP5-GFP at different stages of gametocyte development; GFP fluorescence signal and DIC images (BF). The GFP fluorescence is present in the RBC cytoplasm. *Arrows* in the *top panel* indicate accumulation of PfGEXP5-GFP at the PV. *Scale bars* 5 μm. **c** Immunofluorescence microscopy of sexually induced PfGEXP5-GFP transfectants (14 h pi), probed with rabbit anti-GFP (*green*) and mouse anti-Pfs16 (*red*) antibodies. **d** Immunofluorescence microscopy of sexually induced PfGEXP5-GFP transfectants (14 h pi, *top*) and an asexual trophozoite from the same line (*bottom*), probed with anti-GFP (*green*) and anti-KAHRP (*red*) antibodies. *Scale bars* 3 μm

To investigate PfGEXP5 expression and export in an independent approach, a *P. falciparum* transfectant line was generated expressing full-length PfGEXP5 C-terminally tagged with green fluorescent protein (GFP), autologously regulated by 1.5 kb of the *pfgexp5* genomic upstream region. The parent (3D7) and transfectant (PfGEXP5-GFP) lines were induced to undergo sexual differentiation and stage II–III gametocytes were harvested and selectively lysed with Eqt-II to release the host cell content. Anti-PfGEXP5 antibodies recognized the endogenous protein in the Eqt-II supernatant and an additional band (~60 kDa) corresponding to the PfGEXP5-GFP (Fig. 3a). The relative intensity of the upper band indicates that PfGEXP5-GFP is expressed at a similar level to the endogenous PfGEXP5. Some of the PfGEXP5-GFP chimera is still detectable in the parasite fraction, indicating a less efficient export of the chimera beyond the parasitophorous vacuole (PV), which is not breached by Eqt-II, compared to that of the endogenous protein. When PfGEXP5-GFP transfectants were probed with anti-GFP, only the 62 kDa protein species was detected, with no reactivity in the 3D7 parent (Fig. 3a).

Live cell imaging of the PfGEXP5-GFP transfectant parasites provided further evidence for export of the protein to the host RBC compartment, with the PV as an intermediate compartment (Fig. 3b). In the stage IV and V gametocytes the host cytoplasm is reduced to a thin layer around the parasite, with PfGEXP5-GFP concentrated in this compartment. The persistence of the fluorescence signal in stage V gametocytes may be due to the high stability of the GFP tag.

Live imaging of asexual parasites grown at high parasitaemia to induce gametocytogenesis showed that GFP fluorescence was not detectable in multinucleated parasites, indicating that PfGEXP5 is not expressed in sexually committed schizonts (see Additional file 1: Figure S2B).

Immunofluorescence microscopy confirmed that PfGEXP5-GFP is expressed and exported to the host cell cytoplasm in Pfs16-positive cells (Fig. 3c). In the earliest stages post-induction some cells were observed that were positive for PfGEXP5-GFP but negative for Pfs16 (Fig. 3c), confirming that expression of PfGEXP5 precedes that of Pfs16. No Pfs16-positive/PfGEXP5-GFP-negative parasites were observed, indicating that all stage I gametocytes express PfGEXP5. Consistent with this, the PfGEXP5-GFP positive cells did not exhibit KAHRP labelling at the RBC surface, while KAHRP-positive cells (i.e., asexual stages) were negative for exported PfGEXP5-GFP (Fig. 3d).

These results show that the 1.5 kb of genomic upstream region of the *pfgexp5* gene is sufficient to confer a gametocyte-specific pattern of expression to the PfGEXP5-GFP fusion, and that it contains the *pfgexp5* promoter. The data further indicate that PfGEXP5 is the earliest sexual stage-marker so far described. They also confirm that PfGEXP5 is only expressed in post-invasion sexually committed parasites and is not present at detectable levels in the committed schizonts from the previous cycle.

### PfGEXP5 expression is not under the control of the master regulator of *Plasmodium* sexual differentiation AP2G

The PfGEXP5 marker was exploited to investigate early events in parasite development in the gametocyte defective clones F12 and GNP-A4, in which the mutated PfAP2-G master regulator of sexual differentiation is unable to trigger the expression of 12 early gametocyte transcripts, including those from genes *pfs16* and *pfg27*, and to produce gametocytes [25].

**Fig. 4:**
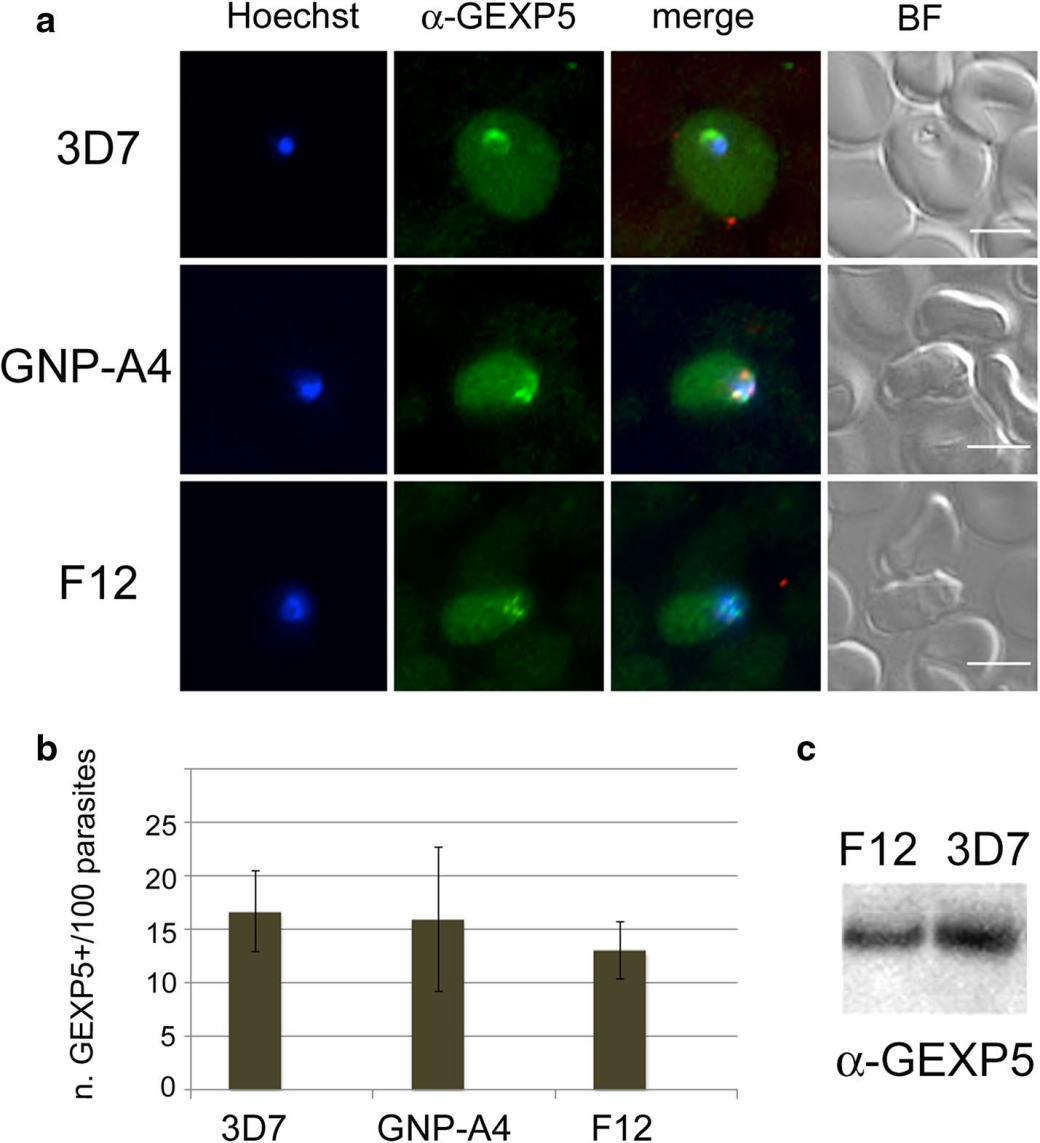
PfGEXP5 expression and export in AP2-G defective parasites. **a** Immunofluorescence microscopy of sexually induced GPN-A4 and F12 parasites at 14 h pi, probed with anti-PfGEXP5 antibodies. *Scale bar* 5 mm. **b** Percent of the above PfGEXP5 positive parasites over total parasites counted at 14 h pi. *Error bars* are standard deviation (from two separate experiments). **c** Western blot analysis with anti-PfGEXP5 antibodies on samples of gametocyte-induced cultures of F12 and 3D7 parasites at 30 h pi

Synchronous F12, GNP-A4 or parental 3D7 parasites were treated using standard gametocyte-inducing conditions. At 12–16 h pi immunofluorescence analysis revealed a sub-population of parasites in each of the three lines that was positive for PfGEXP5 in the host RBC cytoplasm (Fig. 4a). The percentage of PfGEXP5-positive parasites/total parasites (~15 %) was similar in 3D7 and in the two independent AP2G-defective lines (Fig. 4b). Immunofluorescence analysis using anti-Pfg27 at 36 h pi showed that, as expected, no parasite populations became positive for the above early gametocyte marker in F12 and in GNP-A4. In contrast, anti-Pfg27-positive stage I gametocytes were observed in the 3D7 line as expected. At 30 h pi, parasite samples from the F12 and 3D7 cultures were collected, treated with EqtII to release the soluble content of the host RBC and analysed by Western blot. This experiment confirmed that PfGEXP5 was present at comparable levels in the RBC soluble fraction of both parasites (Fig. 4c).

The observation that expression and export of PfGEXP5 in parasites at 14 h pi are comparable irrespective of the presence of a functional AP2G indicates that PfGEXP5 expression is not under control of this regulator. The transcriptional profiles of *pfgexp5* in wild type and in the AP2G-defective F12 parasite line are similar in the period 3–45 h pi (Additional file 1: Figure S3); kindly provided by Manuel Llinas, University of Pennsylvania, USA, from published data [25]). This is consistent with the observation that the PfAP2-G binding site consensus sequence G(T,A,C)GTAC is not present in over 4 kb of the upstream region of the *pfgexp5* gene. These data also indicate that in AP2-G-defective parasites sexual commitment is initiated and then aborted.

## Discussion

### PfGEXP5 is the earliest available marker of *Plasmodium falciparum* gametocyte development

In this work evidence is provided that PfGEXP5 is expressed from 14 h pi in mononucleated, haemozoin-free, ring stage-like parasites that subsequently produce Pfg27. Thus reagents against PfGEXP5 represent potentially valuable tools to investigate very early events in *P. falciparum* sexual differentiation.

Gametocytogenesis is of significant interest as it is the only developmental branch point in the *P. falciparum* life cycle. Gametocyte conversion rates can be modulated in vitro by changing the asexual parasitaemia [5] or otherwise stressing the culture [55, 56], suggesting that the parasite can respond to environmental cues. However an alternative proposal [8] is that a fixed fraction of asexual schizonts commit to gametocytogenesis at every round of asexual replication. As it is virtually impossible to address these questions in the analysis of gametocyte conversion rates in *P. falciparum* natural infections, the availability of a ring stage gametocyte marker will clearly be an invaluable tool.

Several studies have described genes and proteins of possible interest as early gametocyte markers. Transgenic chimeras for several genes expressed in gametocytogenesis have been detected in sub-populations of schizonts, which are assumed to be committed to sexual differentiation. These are *pfg14.748* [30], *pfSET* [57], *pfg27* [36], *pfnek4* [58], *pfgdv1* [8], and *pfAP2*-*G* [25]. However it is not yet clear that all the merozoites expressing these markers develop as gametocytes and continue to produce these proteins very early upon re-invasion. This limits their usefulness as very early sexual markers. Other proteins, such as Pfmdv-1/peg3 and PfPeg4, have been described to appear in early gametocytes, but not in putatively sexually committed schizonts. However, these are expressed at the same time or shortly after Pfs16, Pfg27 [28] and PfGECO [31]. PfGEXP5 is the only protein so far described to be expressed in mononucleated, ring stage-like very early gametocytes from as early as 14 h pi. As the morphology of the PfGEXP5-positive ring stages is indistinguishable from that of asexual ring stages, it is likely that reagents against PfGEXP5 will detect very early gametocytes still in circulation before homing to their sequestration sites. This will provide a breakthrough tool for studies of gametocyte population dynamics in natural infections. It will also enable monitoring of the human infectious reservoirs and will facilitate assessment of the effects of anti-malarial drugs on gametocyte carriage.

### PfGEXP5 expression and host cell remodelling at the onset of gametocytogenesis

The export of the PfGEXP5 and PfGEXP5-GFP into the host cytoplasm was observed, presumably mediated by the PEXEL/HT motif. This is consistent with previous studies showing host cell remodelling in the early phases of gametocyte maturation [29]. Functional roles of exported gametocyte proteins are being elucidated. Disruption of genes PFE1615c and MAL7P1.171, encoding PEXEL/HT-containing proteins, result respectively in failure to produce gametocytes and failure to progress beyond stage I of gametocytogenesis [59]. This work shows that the timing of expression of PfGEXP5 overlaps with that of the cytoskeleton-interacting parasite protein RESA, with the RESA signal waning as the PfGEXP5 signal increases. This provides the first evidence that RESA is expressed in early stage gametocytes, where it may interact with gametocyte-specific proteins. RESA is expressed in the final stages of schizont development, stored in apical organelles within the individual merozoites [60], then transported to the RBC membrane shortly after invasion [61]. Thus its expression may be controlled separately from that of early gametocyte genes.

The *pfgexp5* gene encodes a protein with a conserved PHIST domain, recently classified as belonging to the PHIST subgroup C [48]. Other PHIST C and PHIST B proteins are expressed maximally during schizogony and after invasion in ring stage parasites [62]. The conserved PHIST C domain (a six-helical, segment-structural element that is thought to be involved in protein–protein interactions) may give a clue to its function. PHIST-containing proteins are found only in the genus *Plasmodium* suggesting that the domain evolved after the divergence of the lineage from other Apicomplexa and that the protein family has undergone a dramatic expansion in *P. falciparum* [48, 62]. A similar domain is found in RESA where it may play a role in binding to spectrin and protecting the RBC membrane from damage during shear stress and thermal challenge [63, 64]. Another member of the PHIST family (PF3D7_0402000) binds to protein 4.1, an interaction that appears to lead to dissociation of a sub-population of protein 4.1 molecules from the cytoskeleton and relocation to the PV membrane [65]. These observations lead us to speculate that PfGEXP5 plays a role in re-sculpting the host RBC cytoplasm from a very early stage of gametocyte differentiation.

### PfGEXP5 expression regulation suggests the existence of multiple checkpoints governing *Plasmodium falciparum* sexual differentiation

The genes that control gametocyte development are beginning to be characterized. Recently, a putative transcription factor, PfAP2-G, was identified as a master regulator controlling the switch to sexual stage development. This was based on the observations that PfAP2-G positively regulates the expression of a subset of early gametocyte genes, including *pfs16* and *pfg27*/*25*, and that PfAP2-G-defective parasites fail to produce gametocytes. The observation that such mutants have a selective growth advantage over the wild-type parasites was interpreted as a demonstration that no sexually committed schizonts are produced [25, 66].

Surprisingly, PfGEXP5 is expressed and successfully exported in two clones (F12 and GNP-A4) that are defective for PfAP2-G. This is supported by data from transcriptional analyses of PfAP2-G^+^ and PfAP2-G^−^ parasites indicating that the *pfgexp5* transcript is not upregulated by PfAP2-G at the onset of sexual differentiation [25]. These observations indicate that regulation of *pfgexp5* is most likely governed by a PfAP2-G independent regulatory mechanism.

The fact that sub-populations of F12 and GNP-A4 parasites are able to express and export PfGEXP5 under conditions that normally trigger gametocytogenesis indicates that commitment to sexual development can take place in the absence of functional PfAP2-G. However PfGEXP5 expression is not sufficient to support continued gametocyte development in a background where PfAP2-G is mutated (and thus other early sexual-stage transcripts are not up-regulated). This suggests that multiple mechanisms cooperate to support sexual differentiation. *pfgexp5* expression may act in synergy with the PfAP2-G regulatory cascade, perhaps as an additional checkpoint in commitment or in the earliest steps of gametocyte differentiation.

## Conclusions

The expression and export of PfGEXP5 represent the earliest post-invasion sexual stage marker described to date. This opens the possibility to generate tools able to identify sexually committed ring-stage parasites in natural infections. These could be antibody-based and developed as a rapid diagnostic test or used for immunofluorescence analysis of smears collected from patients. Alternatively, levels of *pfGEXP5* transcript could be monitored using quantitative real time (qRT-PCR), possibly revealing freely circulating early sexual stages. In addition, this early gametocyte marker will facilitate efforts to identify and map the human malaria transmission reservoir in human populations. Finally, the finding that regulation of PfGEXP5 does not depend on the AP2-G master regulator of parasite sexual development suggests that, after sexual commitment, differentiation progresses through multiple checkpoints in the early phase of gametocytogenesis.

## Additional file

Additional file 1
**Figure S1.** Immunofluorescence analysis of 3D7 and HB3 gametocytes with antibodies against PfGEXP5, Pfg27 and GST. **Figure S2.** Analysis of the production of endogenous or GFP-fused PfGEXP5 in asexual stages. **Figure S3.** *pfgexp5* transcriptional profile in PfAP2G− and PfAP2G+ parasites.

## Abbreviations

GEXP5: Gametocyte EXported Protein-5
RBC: red blood cell
KAHRP: knob-associated, histidine-rich protein
RESA: ring-infected erythrocyte surface antigen
PV: parasitophorous vacuole
GFP: green fluorescent protein
EqtII: equinatoxin-II
Glc-NAc: *N*-acetyl-glucosamine
