# Supplementary material for: Specific expression and export of the Plasmodium falciparum Gametocyte EXported Protein-5 marks the gametocyte ring stage
Source: Malar J. 2015 Aug 28;14:334. doi: 10.1186/s12936-015-0853-6 (PMC4552133; doi:10.1186/s12936-015-0853-6)
Supplement: Additional file 1 — Figure S1. Immunofluorescence analysis of 3D7 and HB3 gametocytes with antibodies against PfGEXP5, Pfg27 and GST. Figure S2. Analysis of the production of endogenous or GFP-fused PfGEXP5 in asexual stages. Figure S3. pfgexp5 transcriptional profile in PfAP2G− and PfAP2G+ parasites. [file 12936_2015_853_MOESM1_ESM.pdf]

Supplementary Figures for 'Specific expression and export of the *Plasmodium falciparum* Gametocyte EXported Protein-5 marks the gametocyte ring stage' by Tiburcio, Dixon *et al*.

Supplementary Figure S1.

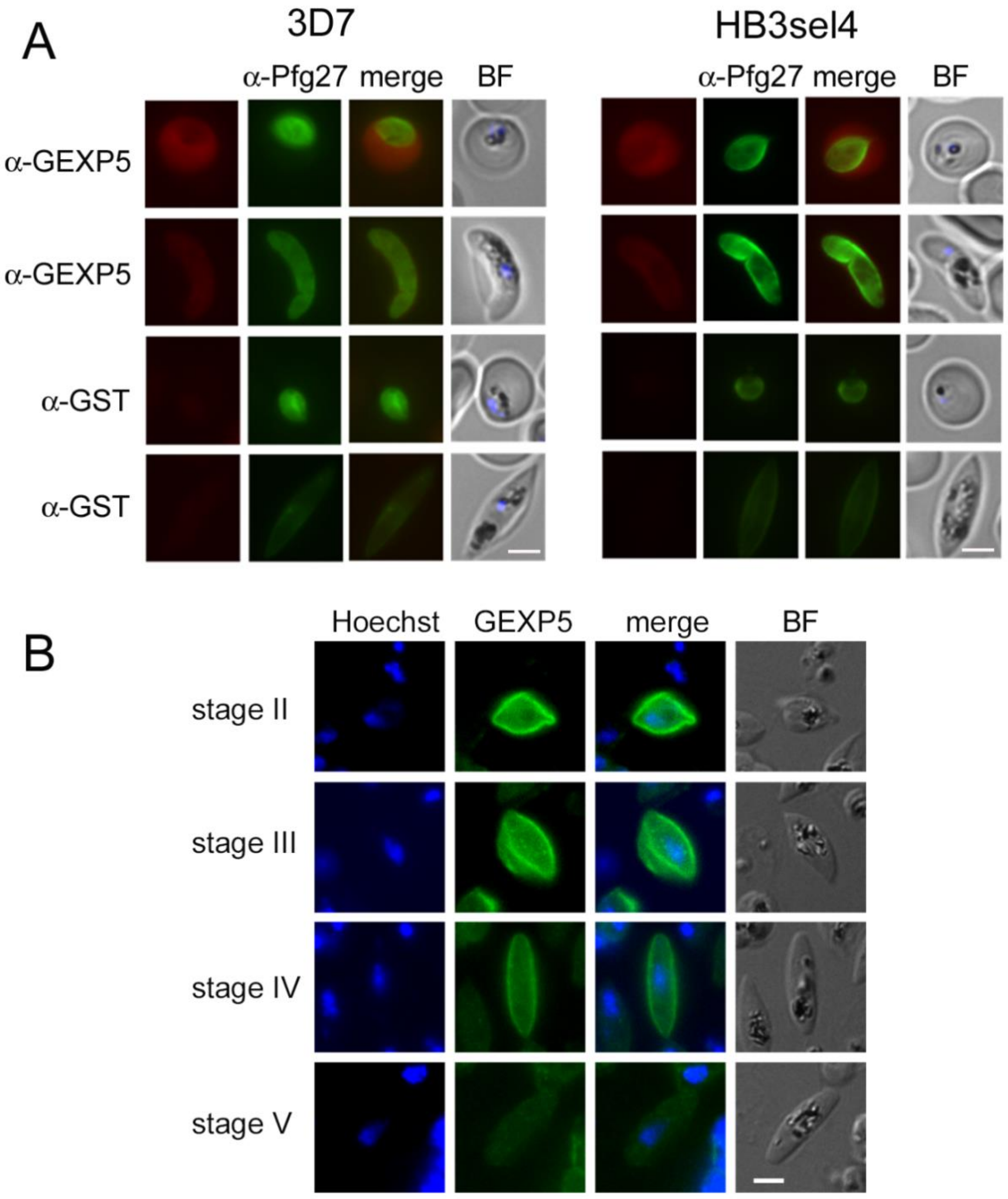

A) Immunofluorescence analysis of stage I (first row) and mature (second row) gametocytes from the genetically distant 3D7 and HB3 lines with mouse anti-PfGEXP5 antibody (red) and rabbit anti-Pfg27 antibody (green). The same stages (third and fourth rows) were also reacted with a rat antibody against GST (red), the moiety of the PfGEXP5 recombinant fusion protein used to obtain the above antibody. No reactivity is detectable on either gametocyte stages with this control antibody. Scale bar is 5 μm. B) Immunofluorescence analysis of stage II to V gametocytes with mouse anti-PfGEXP5 antibody (green). Scale bar is 3 μm.

Supplementary Figure S2.

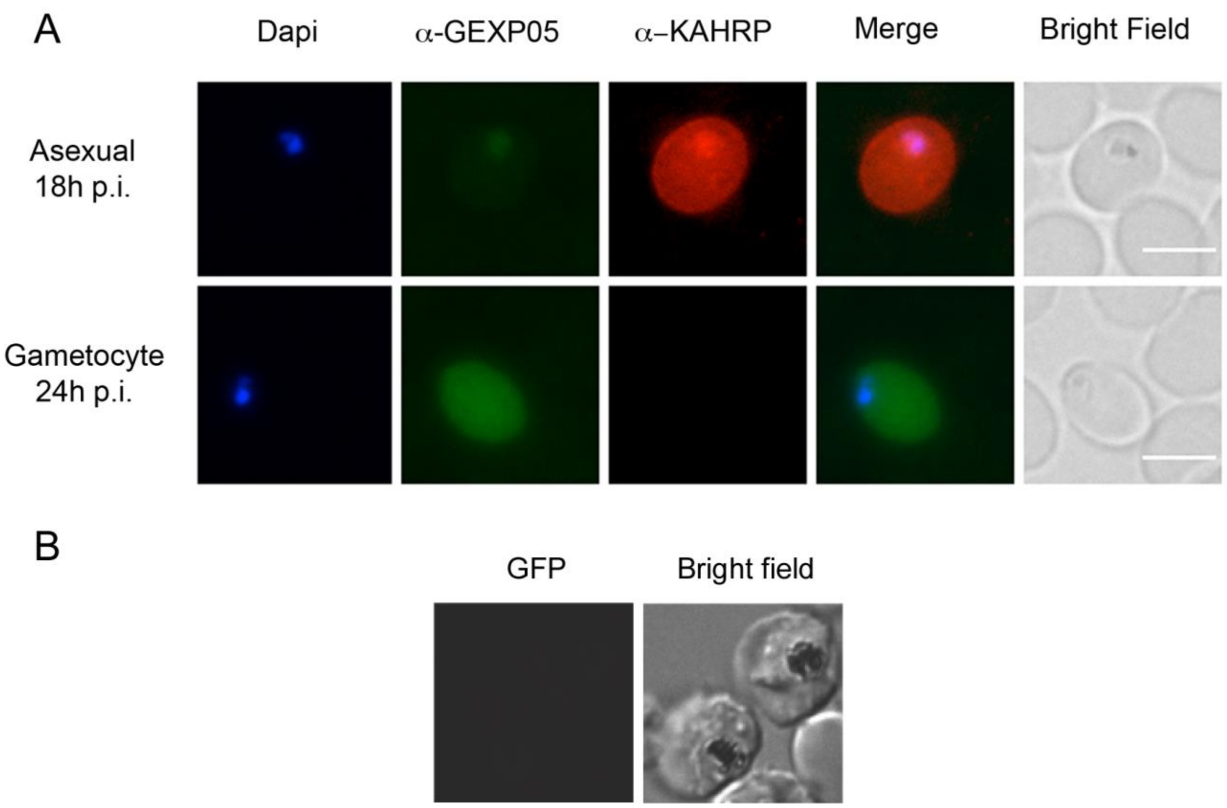

A) Representative immunofluorescence images of (top panel) a GEXP5- negative parasite positively stained by anti-KAHRP antibody (red), and of a GEXP5-positive stage I gametocytes (green), negative for the anti-KAHRP antibody. Parasite line 3D7.

B) Representative image showing that GFP fluorescence is undetectable in schizonts from a sexually induced culture of parasite line 3D7-PfGEXP5-GFP.

Scale bar is 5μm.

Supplementary Figure S3.

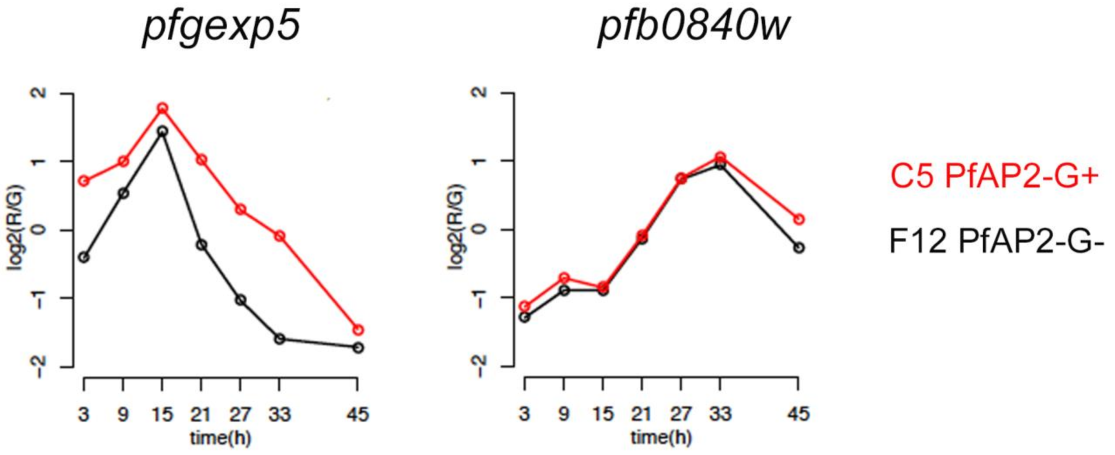

Transcript profiles of the *Pfgexp5* (PFI1770w) and a control gene (PFB0840w) extracted from published microarray data [22]. Courtesy of Manuel Llinas, University of Pennsylvania, USA.
